# Comparison of Three Troponins as Predictors of Future Cardiovascular Events – Prospective Results from the FINRISK and BiomaCaRE Studies

**DOI:** 10.1371/journal.pone.0090063

**Published:** 2014-03-04

**Authors:** Johannes Tobias Neumann, Aki S. Havulinna, Tanja Zeller, Sebastian Appelbaum, Tarja Kunnas, Seppo Nikkari, Pekka Jousilahti, Stefan Blankenberg, Karsten Sydow, Veikko Salomaa

**Affiliations:** 1 Department of General and Interventional Cardiology, Hamburg University Heart Center, Hamburg, Germany; 2 National Institute for Health and Welfare, Department of Chronic Disease Prevention, Helsinki, Finland; 3 Department of Medical Biochemistry, University of Tampere Medical School, Tampere, Finland; 4 German Center for Cardiovascular Research (DZHK), Partner Site Hamburg/Lübeck/Kiel, Germany; Indiana University School of Medicine, United States of America

## Abstract

**Importance and Objective:**

Besides their role in diagnosis of acute myocardial infarction (MI), troponins may be powerful biomarkers for risk stratification in the general population. The objective of our study was to compare the performance of three troponin assays in cardiovascular disease (CVD) risk prediction in a population-based cohort without a history of CVD events.

**Design, Setting and Participants:**

Troponin I concentrations were measured using a contemporary-sensitivity, high-sensitivity, and super-sensitivity assay in 7,899 participants of the general-population based FINRISK 1997 cohort. We used Cox proportional hazards regression to determine relative risks, followed by measures of discrimination and reclassification using 10-fold cross-validation to control for over-optimism.

**Main Outcome:**

As outcome measures we used CVD, MI, ischemic stroke, heart failure (HF), and major adverse cardiac events (MACE). During the follow-up of 14 years 1,074 incident MACE were observed.

**Results:**

Values above the lower limit of detection were observed in 26.4%, 81.5% and 93.9% for the contemporary-sensitivity, high-sensitivity and super-sensitivity assay, respectively. We observed significant associations of troponin concentrations with the risk of future CVD events and the results tended to become stronger with increasing assay sensitivity. For the super-sensitivity assay the multivariate adjusted hazard ratios (per one standard deviation increase) for different outcomes were: MI 1.24 [95% CI 1.11–1.39], stroke 1.14 [1.01–1.28], CVD 1.15 [1.07–1.24], HF 1.28 [1.18–1.39], and MACE 1.18 [1.11–1.25]. In subjects with intermediate risk, we found an improvement of net reclassification for HF (10.2%, p<0.001), and MACE (5.1%, p<0.001).

**Conclusion:**

Using a super-sensitivity assay, cardiac troponin was detectable in almost all healthy individuals. Its concentration improved risk prediction and reclassification for cardiovascular endpoints.

## Introduction

The cardiac-specific protein complex troponin is released in conditions of myocardial damage, and therefore its use as a necrosis marker for the diagnosis of acute myocardial infarction (MI) is well established [1], [2]. Application of sensitive cardiac troponin assays allow an early distinction between acute MI, and other acute cardiac events [3].

Identifying individuals with increased risk for incident cardiovascular diseases (CVD) is a major aim in primary prevention [4]. Thus, the use of biomarkers to identify and monitor individuals at high risk of CVD is emerging [5]. Besides the role in diagnosis of acute cardiac events, troponin T was shown to be a powerful biomarker for risk stratification in individuals with stable atherosclerotic disease [6], [7]. With troponin T assays it is possible to measure troponin concentration in approximately 25% of the general population. Using new high-sensitivity troponin I assays, it is now possible to measure very low troponin concentrations in more than 50% of healthy individuals, and with super-sensitivity troponin I assays in more than 95% of healthy individuals [8]. In patients with stable coronary heart disease, troponin concentrations measured by high-sensitivity assays were significantly associated with cardiovascular death and heart failure [9]. Furthermore, it was shown to be a prognostic marker for MI and cardiovascular death in a population with increased cardiovascular risk [10]. In a population-based setting, concentrations of high-sensitivity assayed troponin were associated with an increased risk for all-cause mortality [8], [11]. In the population-based Framingham Heart Study and the Minnesota Heart Survey troponin concentrations measured by a super-sensitivity troponin assay were significantly associated with major adverse cardiovascular events (MACE), heart failure (HF), and cardiovascular death [12], [13].

The use of more sensitive troponin assays has revealed a high prognostic potential of low troponin concentrations, but their clinical value in risk prediction has not been established and may depend on the sensitivity of the assay in question. Therefore, the objective of our study was to examine the hypothesis that a more sensitive assay detects more individuals at risk of future cardiovascular disease in a population-based cohort without a history of prior MACE. To address this issue, we measured troponin concentrations using a contemporary-sensitivity, high-sensitivity, and super-sensitivity troponin assay in 7,899 participants of the FINRISK 1997 cohort followed up for 14 years.

## Methods

### Study population

The present study included 8,444 individuals from the FINRISK study enrolled in 1997. This prospective population-based study was carried out in five districts of Finland, including North Karelia, Northern Savo (former Kuopio), Southwestern Finland, Oulu province, and the region of Helsinki and Vantaa. A stratified, random sample was drawn from the national population register, the age-range was 25–74 years. All individuals enrolled in the study received a physical examination, a self-administered questionnaire, and a blood sample was drawn. Altogether, 11,500 individuals were invited and 8,444 (73%) participated in the clinical examination. Individuals with a prevalent history of MACE (n = 470) and pregnant women (n = 76) were excluded from the present analysis. During a follow-up of up to 14 years, the National Hospital Discharge Register, the National Causes of Death Register and the National Drug Reimbursement Register were used to identify the endpoints [14]. The design of the FINRISK study has been published before [15]. The Ethics Committee of the National Public Health Institute approved the study, which followed the Declaration of Helsinki. All subjects gave written informed consent.

### Cardiovascular risk factors and diseases

The blood pressure measurement was performed on the right arm in a sitting position after a 5-minute phase of rest. The cuff length was 40 cm. The mean of two measurements was used in the analyses. Data on the use of antihypertensive medications were collected with the questionnaire. Arterial hypertension was defined according to the American Heart Association (AHA) definition, meaning a blood pressure ≥140 mmHg systolic or ≥90 mmHg diastolic, or the use of antihypertensive medication. Smokers were classified by questionnaire as active smokers (smoking regularly ≥1 year and during the past 6 months), former smokers (smoked regularly ≥1 year and quit smoking ≥6 months before the survey), and non-smokers (never smoked regularly). Diabetes was defined either as previously diagnosed diabetes or impaired glucose tolerance by a physician, or by taking any hypoglycemic drugs.

### Defined Endpoints

The follow-up rate was 100% for the participants who continued living in Finland. Those who had permanently moved abroad (0.5% of the participants prior to Dec 31^st^, 2010) were lost to follow-up. The study endpoints were defined as follows. CVD included MI, coronary death, hospitalized unstable angina pectoris, any coronary revascularization, and ischemic stroke. Further endpoints were incident ischemic stroke (hemorrhagic strokes were excluded), incident HF, and MACE (CVD or HF). In a sub-analysis we further distinguished fatal MI from non-fatal MI. The use of Finnish national health care registries for identifying these cardiovascular outcomes has been validated [16].

### Laboratory methods

Prior to drawing the blood samples, the individuals were asked for a 4-hour fasting period, avoiding heavy meals during the day. The median fasting time was 5 hours with an interquartile range of 3–7 hours. The blood samples were stored under standardized conditions at −70°C. Most routine laboratory parameters were measured at the Disease Risk Unit in the National Institute for Health and Welfare, Helsinki. The measurement of CRP, N-terminal pro-brain natriuretic peptide (NT-proBNP), and the different troponin assays were performed at the MORGAM Biomarker Laboratory, University Heart Center Hamburg, Germany, which was formerly located at the University Medical Center Mainz, Germany. The contemporary-sensitivity troponin I assay (STAT troponin I immunoassay, Abbott Diagnostics, USA; ARCHITECT i2000SR) was considered valid for values above the limit of detection (LOD) of 10 pg/mL, but observed values below this limit were also used in the analysis (assay range 0–50,000 pg/mL). The 10% coefficient of variation was at 32 pg/mL. Troponin was also assessed using a prototype high-sensitivity cardiac troponin assay (ARCHITECT STAT highly sensitive troponin I immunoassay, Abbott Diagnostics, USA, ARCHITECT i2000SR). The established LOD for the assay ranges from 0.8–1.9 with a median of 1.5 pg/mL. For analyses, a LOD of 1.9 pg/mL was considered. Observed values below this limit were also included in the analysis (assay range 0–50,000 pg/mL). The 10 percent coefficient of variation was at 5.2 pg/mL. The concentration representing the 99^th^ percentile in the reference population was 30 pg/mL in 4,139 individuals of the population-based Gutenberg Health Study [1]. The super-sensitivity troponin I (Erenna Cardiac troponin-I immunoassay, Singulex, USA) had a median LOD of 1.0 pg/mL and an assay range of 0.1–600 pg/mL. The 10% coefficient of variation was between 0.78 and 1.6 pg/mL [17]. Again, values below the LOD were included in the analysis.

### Statistical analysis

Baseline characteristics are presented as counts and percentages for dichotomous variables, and as median and IQR for continuous variables. Age and sex adjusted Kaplan-Meier-curves for MACE and HF were produced using categorized troponin concentrations. For the contemporary-sensitivity assay, we used 1 pg/mL (lowest observed non-zero value) and 10 pg/mL (assay threshold) as cut-points. For the high-sensitivity assay, we used 1.9 pg/mL (assay threshold), and 5.1 pg/mL (same percentile as 10 pg/mL for the contemporary assay) as cut-points. We proceeded the same way for the super-sensitivity assay, using cut-points of 1.0 pg/mL (median limit of detection) and 5.1 pg/mL (same percentile as 10 pg/mL for the contemporary assay). To describe the association of troponin with clinical endpoints, Cox regression models adjusting for the variables of the Framingham risk score (log-transformed total cholesterol, log-transformed HDL, log-transformed systolic blood pressure, hypertension medication, diabetes, current smoking) and region of Finland (east, west) were computed. We used the Framingham equation meant for estimating the 10-year risk of cardiovascular event in primary care [18]. In these analyses troponin concentrations were log-transformed, and age was used as the time scale. Multiple imputation techniques were used to manage missing values [19], [20]. The additional value of troponin concentration to the Framingham risk score was assessed by means of the C-index, integrated discrimination improvement (IDI) and net reclassification improvement (NRI) with risk categories [0–5%), [5–10%), [10–20%), and [20–100%] for 10-year risk [21], [22]. The clinical NRI refers to individuals with an intermediate 10-year risk (5–20%) according to the Framingham Risk Score. Ten-fold cross-validation was used to control for over-optimism. R version 15.1 (R Foundation for Statistical Computing, Vienna, Austria) was used for all analyses. All tests were two-tailed and p<0.05 was considered statistically significant. The results of the contemporary-sensitivity troponin I assay in this study population have been published before [19]. In comparison to the earlier publication we now have 3 more years of follow-up with 232 more CVD events. Furthermore, the modeling is slightly different: in the present manuscript the included covariates are different, the troponin I values are log-transformed (instead of cubic root transformed) and the results are cross-validated in the same cohort (instead of a separate validation cohort).

## Results

### Baseline characteristics of the study sample

The FINRISK 1997 study enrolled 7,899 individuals (50.3% women and 49.7% men) after exclusion of those with prevalent MACE and pregnant women (*Table 1*). The mean age of included persons was 47.8 years. The mean baseline concentration of troponin was 3.9 pg/mL when measured with the contemporary-sensitivity assay, 3.0 pg/mL when measured with the high-sensitivity assay, and 2.8 pg/mL when measured with the super-sensitivity assay. Troponin values above the assay thresholds were observed in 26.4% for the contemporary-sensitivity assay, in 81.5% for the high-sensitivity assay, and in 93.9% for the super-sensitivity assay (*Table 2*). During the 14 years of follow-up 810 individuals died. All incident MACE accounted to 1,074, which included 299 ischemic strokes, 770 CVD events, and 505 incident heart failures. See Table 1 for further details of the baseline characteristics.

**Table 1:** Baseline Characteristics of the Study Population by Gender.

	All (N = 7899)	Women (N = 3970, 50.3%)	Men (N = 3929, 49.7%)
Age (years)	47.8 (21.8)	46.9 (21)	48.8 (22.5)
**Cardiovascular risk factors**			
Current smoker (%)	2060 (26.1%)	817 (20.6%)	1243 (31.6%)
Former smoker (%)	1637 (20.7%)	555 (14%)	1081 (27.5%)
Hypertension (%)	3522 (44.6%)	1461 (36.8%)	2061 (52.5%)
Diabetes (%)	403 (5.1%)	188 (4.7%)	215 (5.5%)
**Clinical parameters**			
Body mass index (kg/m^2^)	26.0 (5.5)	25.4 (6.3)	26.5 (4.8)
Systolic BP (mmHg)	133 (27)	129 (26)	136 (25)
Total Cholesterol (mmol/L)	5.5 (1.4)	5.4 (1.4)	5.5 (1.4)
HDL Cholesterol (mmol/L)	1.4 (0.5)	1.5 (0.5)	1.2 (0.4)
**Biomarkers**			
cs-cTnI (pg/mL)	3.9 (9.0)	3.6 (9.7)	4.1 (7.8)
hs-cTnI (pg/mL)	3.0 (2.6)	2.5 (2.1)	3.6 (3.0)
ss-cTnI (pg/mL)	2.8 (3.0)	2.5 (2.6)	3.2 (3.2)
CRP (mg/L)	1.1 (1.9)	1.1 (1.9)	1.1 (1.8)
NT-proBNP (pg/mL)	42.1 (59.0)	55.0 (61.2)	28.7 (48.4)
**Incident Outcome**			
All-cause death (%)	810 (10.3%)	258 (6.5%)	552 (14%)
Fatal MI (%)	86 (1.1%)	22 (0.6%)	64 (1.6%)
MI (%)	277 (3.5%)	69 (1.7%)	208 (5.3%)
Stroke (%)	299 (3.8%)	93 (2.3%)	206 (5.2%)
Heart failure (%)	505 (6.4%)	221 (5.6%)	284 (7.2%)
CVD (%)	770 (9.8%)	217 (5.5%)	553 (14.1%)
MACE (%)	1074 (13.6%)	372 (9.4%)	702 (17.9%)

Persons with prevalent MACE and pregnant women have been excluded. Binary variables are shown in absolute counts and percentages. For continuous variables the median and the IQR are shown.

MI = Myocardial infarction, MACE = major adverse cardiac events, BP = blood pressure, HDL = high-density-lipoprotein, CRP = c-reactive protein, NT-proBNP = N-terminal pro-brain natriuretic peptide, IQR = interquartile range, CVD = cardiovascular disease, cs-cTnI = troponin I measured by contemporary-sensitivity assay, hs-cTnI = troponin I measured by high-sensitivity assay, ss-cTnI = troponin measured by super-sensitivity assay

**Table 2:** Absolute and Relative Distributions of Troponin I Concentrations in the Study Population.

Troponin	Category 1	Category 2	Category 3	% above LOD
cs-cTnI	2273	3543	2083	26.4%
hs-cTnI	1462	4653	1784	81.5%
ss-cTnI	479	5641	1779	93,9%

The categories were defined as follows. For the contemporary-sensitivity assay, troponin category 1 is 0–1 pg/mL (lowest observed non-zero value), category 2 is 1–10 pg/mL (LOD), and category 3 is >10 pg/mL. For the high-sensitivity assay, troponin category 1 is 0–1.9 pg/mL (LOD), category 2 1.9–5.1 pg/mL (same percentile as 10 pg/mL for contemporary troponin), and category 3 is >5.1 pg/mL. For the super-sensitivity assay, troponin category 1 is 0–1.0 pg/mL (median limit of detection), category 2 1.0–5.1 pg/mL (according to the percentiles of contemporary troponin), and category 3 is >5.1 mg/mL.

cs-cTnI = troponin I measured by contemporary-sensitivity assay, hs-cTnI = troponin I measured by high-sensitivity assay, ss-cTnI = troponin I measured by super-sensitivity assay, LOD = limit of detection.

### Association of troponin concentration measured by contemporary-sensitivity, high-sensitivity, and super-sensitivity assay with cardiovascular outcome

**Figure 1:**
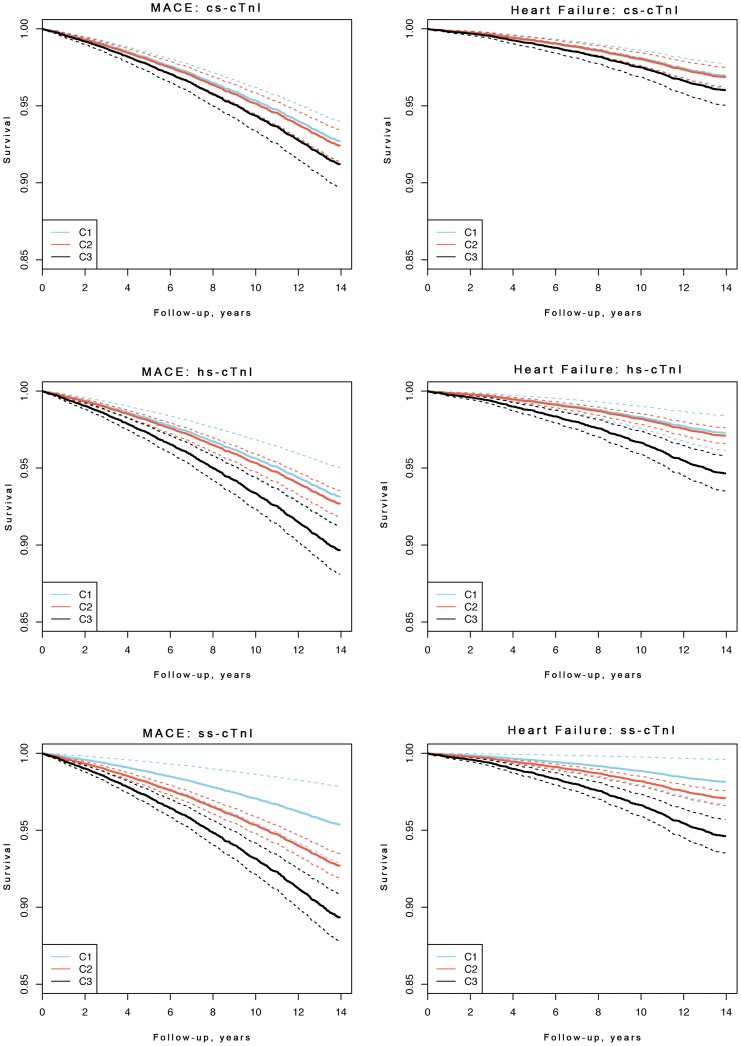
Kaplan-Meier Curves According to Troponin Categories as Measured by Contemporary-Sensitivity, High-Sensitivity and Super-Sensitivity Assays for MACE and HF after Adjustment for Age and Gender. Dotted lines indicate the 95% confidence intervals. MACE = major adverse cardiac events, cs-cTnI = troponin I measured by contemporary-sensitivity assay, hs-cTnI = highly sensitive troponin I measured by high-sensitivity assay, ss-cTnI = troponin I measured by super-sensitivity assay. Please see the footnote to Table 2 for the cut points of the troponin categories.

**Figure 2:**
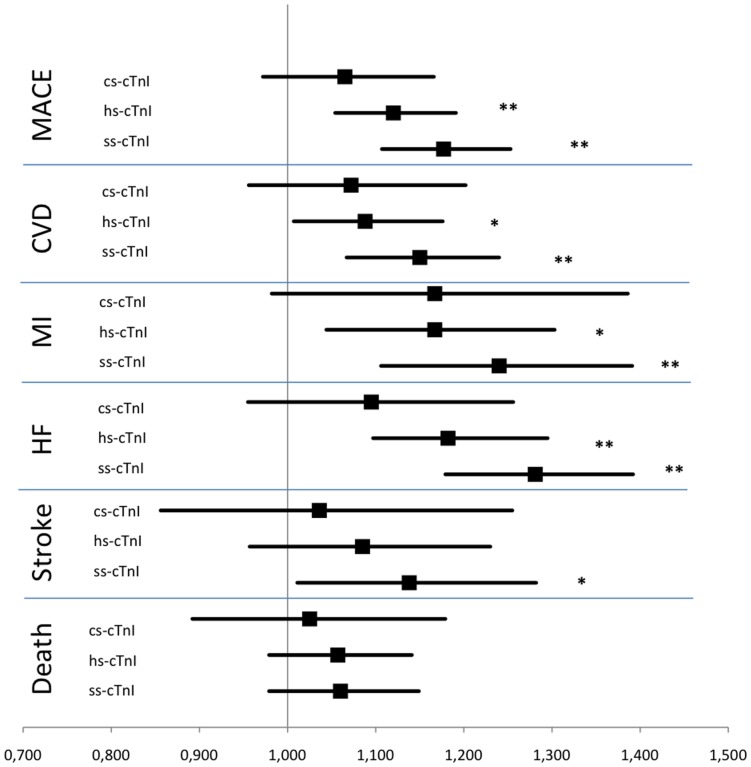
Hazard Ratios from Cox Regression Models for Baseline Troponin Assessed by Contemporary-Sensitivity, High-Sensitivity and Super-Sensitivity Assays for Various Endpoints after Adjustment for the Framingham Risk Score. ** = p value<0.001, * = p value<0.05. MACE = major adverse cardiac events, CVD = cardiovascular disease, MI = myocardial infarction, HF = heart failure, HR = hazard ratio, CI = confidence interval, cs-cTnI = troponin I measured by contemporary-sensitivity assay, hs-cTnI = troponin I measured by high-sensitivity assay, ss-cTnI = troponin I measured by super-sensitivity assay.

Figure 1 presents Kaplan- Meier estimates for MACE and HF, adjusted for age and sex, showing higher survival rates for the lowest troponin category of the supersensitive assay. In the Cox regression model, hazard ratios of the high-sensitivity and the super-sensitivity assays were significantly increased for incident MACE 1.12 [95% CI 1.05–1.19] and 1.18 [1.11–1.25], MI 1.17 [1.04–1.30] and 1.24 [1.11–1.39], and HF 1.19 [1.1–1.3] and 1.28 [1.18–1.39] for continuous values per 1-SD increase (*Table 3* *and* *Fig. 2*). For strokes, only troponin assessed by the super-sensitive assay showed a significantly increased HR 1.14 [1.01–1.28]. No association was seen with all-cause death. Comparing the gender-specific HR, we observed similar values in both sexes (*Table S1 in file S1*). Both, C-reactive protein (CRP) and NT-proBNP, are well-established biomarkers for cardiovascular risk prediction. To control for these variables, we performed an additional adjustment for CRP and NT-proBNP. In this analysis HRs became weaker using the super-sensitivity assay, but were still significant: for MACE 1.11 [1.04–1.19], CVD 1.1 [1.02–1.19], MI 1.19 [CI 1.05–1.34], and HF 1.18 [1.08–1.29] (*Table S3 in file S1*). Troponin assessed by the high-sensitivity assay showed a significantly increased HR for MI 1.12 [CI 1.0–1.26], and HF 1.11 [1.01–1.21]. When considering categorical values, HRs were generally higher for the highest troponin category than the lowest, but a significantly increased HR was observed only for HF (high-sensitivity and super-sensitivity assay) and MACE (super-sensitivity assay) (*Table S2 in file S1*). The use of the contemporary-sensitivity and super-sensitivity troponin assays improved the discrimination beyond the Framingham Risk Model for CVD and MI (*Table S3 in file S1*). Troponin measurement using the high-sensitivity and super-sensitivity troponin assays improved discrimination for MACE and HF, while no significant improvement was seen for stroke and death.

**Table 3:** Hazard Ratios from Cox Regression Models for Baseline Troponin Assessed by Contemporary-Sensitivity, High-Sensitivity and Super-Sensitivity Assays for Various Endpoints after Adjustment for the Framingham Risk Score.

		Continuous		Categorical	
Event	Troponin	HR (95% CI)	p value	HR (95% CI)	p value
**MACE**	cs-cTnI	1.07 (0.97–1.17)	ns	1.22 (0.95–1.57)	ns
	hs-cTnI	1.12 (1.05–1.19)	<0.001	1.24 (0.91–1.69)	ns
	ss-cTnI	1.18 (1.11–1.25)	<0.001	2.10 (1.21–3.63)	0.008
**CVD**	cs-cTnI	1.07 (0.96–1.20)	ns	1.20 (0.87–1.64)	ns
	hs-cTnI	1.09 (1.01–1.18)	0.032	1.08 (0.74–1.58)	ns
	ss-cTnI	1.15 (1.07–1.24)	<0.001	1.95 (0.97–3.93)	ns
**MI**	cs-cTnI	1.17 (0.10–1.39)	ns	1.52 (0.90–2.57)	ns
	hs-cTnI	1.17 (1.04–1.30)	0.006	1.11 (0.59–2.10)	ns
	ss-cTnI	1.24 (1.11–1.39)	<0.001	2.85 (0.69–11.68)	ns
**HF**	cs-cTnI	1.10 (0.96–1.26)	ns	1.31 (0.90–1.89)	ns
	hs-cTnI	1.19 (1.10–1.30)	<0.001	1.68 (1.04–2.72)	0.036
	ss-cTnI	1.28 (1.18–1.39)	<0.001	2.59 (1.15–5.82)	0.02
**Stroke**	cs-cTnI	1.04 (0.86–1.26)	ns	1.02 (0.63–1.65)	ns
	hs-cTnI	1.09 (0.96–1.23)	ns	0.99 (0.55–1.78)	ns
	ss-cTnI	1.14 (1.01–1.28)	0.032	1.97 (0.64–6.09)	ns
**Death**	cs-cTnI	1.03 (0.89–1.18)	ns	1.04 (0.73–1.47)	ns
	hs-cTnI	1.06 (0.98–1.14)	ns	1.40 (0.96–2.05)	ns
	ss-cTnI	1.06 (0.98–1.15)	ns	1.02 (0.64–1.64)	ns

Shown are the hazard ratios for continuous troponin concentration per 1-SD increment and for categorical troponin concentration, comparing the highest with the lowest defined category. Please see the footnote to Table 2 for cut points of the categories.

Abbreviations: MACE = major adverse cardiac events, CVD = cardiovascular disease, MI = myocardial infarction, HF = heart failure, HR = hazard ratio, CI = confidence interval, cs-cTnI = troponin I measured by contemporary-sensitivity assay, hs-cTnI = highly sensitive troponin, ss-cTnI = supersensitive troponin, ns = not significant.

### Risk reclassification by determination of baseline troponin concentration

Net reclassification for the different events did not improve in the overall analysis (*Table 4*). Focusing on individuals with an intermediate 10-year risk (5–20%) according to the Framingham Risk Score, we determined the clinical NRI. The strongest clinical NRI for incident CVD was achieved with inclusion of troponin measured with the super-sensitivity assay followed by the troponin measured with the contemporary-sensitivity assay (3.46%, p = 0.009 and 2.30%, p = 0.026). Considering HF and MACE we observed a significantly improved reclassification (HF 10.23%, p<0.001; MACE 5.1%, p<0.001), when using the super-sensitivity troponin assay.

**Table 4:** Net Reclassification Improvement (NRI) and Clinical NRI for Various Endpoints for Baseline Troponin Assessed by Contemporary-Sensitivity, High-Sensitivity and Super-Sensitivity Assays in Addition to the Standard Framingham Risk Score.

Event	Troponin	NRI	p value	Clinical NRI	p value
**MACE**	Cs-cTnI	0.22%	ns	1.86%	0.029
	hs-cTnI	−0.08%	ns	2.63%	0.016
	ss-cTnI	0.28%	ns	5.13%	<0.001
**CVD**	cs-cTnI	0.69%	ns	2.30%	0.026
	hs-cTnI	−0.14%	ns	1.70%	ns
	ss-cTnI	0.48%	ns	3.46%	0.009
**MI**	cs-cTnI	3.37%	ns	4.81%	ns
	hs-cTnI	2.90%	ns	3.95%	ns
	ss-cTnI	5.10%	ns	7.50%	ns
**HF**	cs-cTnI	1.03%	ns	2.19%	ns
	hs-cTnI	2.85%	ns	6.97%	0.004
	ss-cTnI	4.09%	ns	10.23%	<0.001
**Stroke**	cs-cTnI	0.02%	ns	0.27%	ns
	hs-cTnI	1.24%	ns	−0.04%	ns
	ss-cTnI	−0.80%	ns	−2.10%	ns
**Death**	cs-cTnI	0.09%	ns	0.70%	ns
	hs-cTnI	−0.40%	ns	0.02%	ns
	ss-cTnI	0.35%	ns	2.66%	0.023

NRI = net reclassification improvement, clinical NRI = NRI for individuals with an intermediate 10-year risk (5–20%) according to the Framingham Risk Score, MACE = major adverse cardiac events, CVD = cardiovascular disease, MI = myocardial infarction, HF = heart failure, cs-cTnI = troponin I measured by contemporary-sensitivity assay, hs-cTnI = troponin I measured by high-sensitivity assay, ss-cTnI = troponin I measured by super-sensitivity assay, ns = not significant.

## Discussion

The use of more sensitive troponin assays has revealed a high potential for diagnosis of acute cardiac events, but also for identifying individuals at risk of a future cardiovascular event. This study compared the predictive power of baseline troponin concentrations in a population-based setting using three troponin assays of different sensitivity, ranging from a contemporary-sensitivity and high-sensitivity to a super-sensitivity assay, in individuals without a history of MACE. We observed a generally stronger association of troponin concentrations with MACE, CVD, MI, HF, and stroke, when the super-sensitivity assay was used compared to the high-sensitivity and contemporary-sensitivity assays. The associations remained significant after adjustment for the classical risk factors included in the Framingham equation, and additional adjustment for CRP and NT-proBNP for the endpoints MACE, CVD, MI, and HF. Net reclassification improvement was not significant among all subjects. In subjects with intermediate risk, however, reclassification was significantly improved for various endpoints. The strongest clinical reclassification improvement was found with the super-sensitivity troponin assay for MACE and HF.

Previous studies have shown that troponin predicts cardiovascular events in the general population [8]. In the Dallas Heart Study, troponin T was detectable with a high-sensitivity assay in 25% of the general population and significantly associated with structural heart disease and mortality [8]. In older patients of the Cardiovascular Health Study, troponin T was measured by a high-sensitivity assay with a detection rate of 66%. Detectable troponin T concentrations were associated with incident HF and cardiovascular death [23]. In the Atherosclerosis Risk in Communities Study high-sensitivity assayed troponin T was also measurable in 66% of the study population and associated with CHD, mortality, and HF [24]. In contrast to these findings, detection rates in the present study were higher, since troponin I measured by the high-sensitivity and super-sensitivity assay was above the assay threshold in 82% and in 94% of individuals, respectively. In a recently published overview on 19 different troponin assays, the same high-sensitivity and super-sensitivity troponin I assays were used and showed even higher detection rates (96% and 100%, respectively) when using newer assays with lower LODs [25]. The same super-sensitivity assay was used in the Minnesota Heart Survey and showed a significant association with cardiovascular death [13]. These results are confirmed by our current findings with much larger material, and extended to include non-fatal endpoints. The troponin overview by Apple showed important differences in the 99^th^ percentile according to gender with mostly higher concentrations in males. In our study median troponin concentrations measured by either the high-sensitivity or the super-sensitivity assay were higher in males. The measured HRs however did not differ significantly. Nevertheless gender-specific analysis should be considered for the interpretation of absolute troponin concentrations. Furthermore, recent results from the Scottish Heart Health Extended Cohort study suggested that the optimal (in terms of sensitivity and specificity) cutpoints of high-sensitivity troponin I differed between men and women [26]. The supersensitive troponin I was, however, not determined in the Scottish study. With the super-sensitivity troponin assay it is possible to measure very low troponin concentrations, reflecting minor myocardial processes without major ischemic damage. Therefore the use of super-sensitivity troponin assays apparently enables an improvement of risk prediction in the general population, whereas its superiority in settings of acute coronary syndrome diagnosis needs to be further elucidated.

A particular strength of the present study is the comparison of three troponin assays with different sensitivities in risk prediction. However, various limitations merit consideration. First, measurements have been performed in frozen samples that were stored for 14 years at −70°C and had been thawed twice. Second – and most important – we do not have sufficient data at the moment to fully understand the clinical consequences of reclassification in those individuals reclassified from the intermediate risk group to the high-risk group. A recent report suggested medical intervention with statin therapy after such reclassification obtained using measurements of fibrinogen or CRP [27]. That paper demonstrated the potential of the approach but the reclassification with CRP or fibrinogen was relatively modest. The present study and our earlier work suggest that better reclassification can be obtained with troponin or by combining troponin with other biomarkers into a biomarker score [19]. It seems, however, that a randomized clinical trial would be needed to unequivocally demonstrate the benefits and risks of this approach. In contrast to our earlier work the contemporary-sensitivity assay was not able to predict outcome in the present study [19]. This is explained by the longer follow-up period, the larger number of CVD events and somewhat different modelling strategy. Nevertheless, the HRs of our earlier study and the present study are close to each other and the 95% CIs are widely overlapping. Finally, this study is limited to the Finnish population. Therefore, regional and especially ethnic differences of the measured biomarkers might have been missed.

### Conclusion

Using a super-sensitivity assay, troponin I was detectable in almost all healthy individuals in this population-based setting. In terms of relative risk, troponin I was a significant predictor for future CVD events over and above the standard Framingham equation and remained significant even after further adjustment for CRP and NT-proBNP. In subjects at intermediate risk of CVD, the super-sensitivity troponin assay significantly improved reclassification for HF and MACE outcomes. Although both, the super-sensitivity and the high-sensitivity assays are predictors for incident CVD, application of the super-sensitivity assay might hold a slightly higher potential to reclassify the outcome.

## Supporting Information

File S1 **File containing Tables S1–S3.** Table S1: Hazard Ratios from Cox Regression Models for Baseline Troponin Assessed by Contemporary-Sensitivity, High-Sensitivity and Super-Sensitivity Assays for Various Endpoints by Gender after Adjustment for the Framingham Risk Score. Table S2: Hazard Ratios from Cox Regression Models for Baseline Troponin Assessed by Contemporary-Sensitivity, High-Sensitivity and Super-Sensitivity Assays for Various Endpoints, after Adjustment for Framingham Risk Score, CRP and NT-proBNP. Table S3: C-Statistics and Integrated Discrimination Improvement for Baseline Troponin Assessed by Contemporary-Sensitivity, High-Sensitivity and Super-Sensitivity Assays for Various Endpoints after Adjustment for the Framingham Risk Score.
(DOC)
