# Supplementary material for: Comparison of Three Troponins as Predictors of Future Cardiovascular Events – Prospective Results from the FINRISK and BiomaCaRE Studies
Source: PLoS One. 2014 Mar 4;9(3):e90063. doi: 10.1371/journal.pone.0090063 (PMC3942371; doi:10.1371/journal.pone.0090063)
Supplement: File S1 — File containing Tables S1–S3. Table S1: Hazard Ratios from Cox Regression Models for Baseline Troponin Assessed by Contemporary-Sensitivity, High-Sensitivity and Super-Sensitivity Assays for Various Endpoints by Gender after Adjustment for the Framingham Risk Score. Table S2: Hazard Ratios from Cox Regression Models for Baseline Troponin Assessed by Contemporary-Sensitivity, High-Sensitivity and Super-Sensitivity Assays for Various Endpoints, after Adjustment for Framingham Risk Score, CRP and NT-proBNP. Table S3: C-Statistics and Integrated Discrimination Improvement for Baseline Troponin Assessed by Contemporary-Sensitivity, High-Sensitivity and Super-Sensitivity Assays for Various Endpoints after Adjustment for the Framingham Risk Score. (DOC) [file pone.0090063.s001.doc]

**SUPPLEMENTAL MATERIAL TO**

**Comparison of Three Troponins as Predictors of Future Cardiovascular Events – Prospective Results from the FINRISK Study**

Johannes Tobias Neumann^1^; Aki S. Havulinna^2^; Tanja Zeller^1^; Sebastian Appelbaum^1^; Tarja Kunnas^2^; Seppo Nikkari^2^; Pekka Jousilahti^2^; Stefan Blankenberg^1^; Karsten Sydow^1^; Veikko Salomaa^2^

^1^ Department of General and Interventional Cardiology, Hamburg University Heart Center, Hamburg, Germany

^2^ National Institute for Health and Welfare, Department of Chronic Disease Prevention, Helsinki, Finland

**Corresponding Author:**

Veikko Salomaa, MD PhD
Department of Chronic Disease Prevention
National Institute for Welfare and Health
Pl 30, FI-00271 Helsinki, Finland
Phone: +358-20 6108620
E-mail: veikko.salomaa@thl.fi

**Supplemental Material**

**Table S**1: Hazard Ratios from Cox Regression Models for Baseline Troponin Assessed by Contemporary-Sensitivity, High-Sensitivity and Super-Sensitivity Assays for Various Endpoints by Gender after Adjustment for the Framingham Risk Score

|  |  | **Female** | | **Male** | |
| --- | --- | --- | --- | --- | --- |
| **Event** | **Troponin** | **HR (95% CI)** | **p value** | **HR (95% CI)** | **p value** |
| **MACE** | cs-cTnI | 0.94 (0.79–1.13) | ns | 1.13 (1.02–1.25) | 0.024 |
|  | hs-cTnI | 1.13 (1.02–1.26) | 0.018 | 1.11 (1.03–1.20) | 0.005 |
|  | ss-cTnI | 1.23 (1.11–1.36) | <0.001 | 1.14 (1.06–1.23) | <0.001 |
| **CVD** | cs-cTnI | 0.95 (0.77–1.20) | ns | 1.11 (0.98–1.26) | ns |
|  | hs-cTnI | 1.10 (0.95–1.28) | ns | 1.08 (0.99–1.18) | ns |
|  | ss-cTnI | 1.16 (1.01–1.33) | 0.042 | 1.13 (1.04–1.23) | 0.004 |
| **MI** | cs-cTnI | 1.21 (0.98–1.67) | ns | 1.14 (0.93–1.40) | ns |
|  | hs-cTnI | 1.19 (0.96–1.47) | ns | 1.17 (1.03–1.32) | 0.019 |
|  | ss-cTnI | 1.16 (0.91–1.48) | ns | 1.25 (1.11–1.41) | <0.001 |
| **HF** | cs-cTnI | 0.94 (0.72–1.22) | ns | 1.22 (1.06–1.41) | 0.007 |
|  | hs-cTnI | 1.12 (0.98–1.27) | ns | 1.24 (1.12–1.38) | <0.001 |
|  | ss-cTnI | 1.29 (1.13–1.47) | <0.001 | 1.26 (1.14–1.40) | <0.001 |
| **Stroke** | cs-cTnI | 0.90 (0.60–1.34) | ns | 1.09 (0.89–1.33) | ns |
|  | hs-cTnI | 1.10 (0.88–1.38) | ns | 1.07 (0.93–1.24) | ns |
|  | ss-cTnI | 1.20 (0.97–1.47) | ns | 1.10 (0.96–1.26) | ns |
| **Death** | cs-cTnI | 1.03 (0.81–1.32) | ns | 1.01 (0.87–1.18) | ns |
|  | hs-cTnI | 1.11 (0.978–1.25) | ns | 1.03 (1.00–1.41) | 0.049 |
|  | ss-cTnI | 1.06 (0.93–1.21) | ns | 1.06 (0.96–1.16) | ns |

MACE = major adverse cardiac events, CVD = cardiovascular disease, MI = myocardial infarction, HF = heart failure, HR = hazard ratio, CI = confidence interval, cs-cTnI = troponin I measured by contemporary-sensitivity assay, hs-cTnI = troponin I measured by high-sensitivity assay, ss-cTnI = troponin I measured by super-sensitivity assay, ns = not significant

**Table S**2: Hazard Ratios from Cox Regression Models for Baseline Troponin Assessed by Contemporary-Sensitivity, High-Sensitivity and Super-Sensitivity Assays for Various Endpoints, after Adjustment for Framingham Risk Score, CRP and NT-proBNP

| **Event** | **Troponin** | **HR (95% CI)** | **p value** |
| --- | --- | --- | --- |
| **MACE** | cs-cTnI | 1.02 (0.93–1.13) | ns |
|  | hs-cTnI | 1.06 (1.00–1.14) | ns |
|  | ss-cTnI | 1.11 (1.04–1.19) | 0.001 |
| **CVD** | cs-cTnI | 1.04 (0.92–1.17) | ns |
|  | hs-cTnI | 1.04 (0.96–1.13) | ns |
|  | ss-cTnI | 1.10 (1.02–1.19) | 0.019 |
| **MI** | cs-cTnI | 1.12 (0.94–1.34) | ns |
|  | hs-cTnI | 1.12 (1.00–1.26) | 0.054 |
|  | ss-cTnI | 1.19 (1.05–1.34) | 0.005 |
| **HF** | cs-cTnI | 1.03 (0.89–1.20) | ns |
|  | hs-cTnI | 1.11 (1.01–1.21) | 0.026 |
|  | ss-cTnI | 1.18 (1.08–1.29) | <0.001 |
| **Stroke** | cs-cTnI | 1.00 (0.81–1.21) | ns |
|  | hs-cTnI | 1.02 (0.89–1.17) | ns |
|  | ss-cTnI | 1.07 (0.94–1.21) | ns |
| **Death** | cs-cTnI | 0.99 (0.86–1.14) | ns |
|  | hs-cTnI | 1.01 (0.93–1.10) | ns |
|  | ss-cTnI | 1.02 (0.93–1.10) | ns |

CRP = c-reactive protein, NT-proBNP = brain-natriuretic peptide, MACE = major adverse cardiac events, CVD = cardiovascular disease, MI = myocardial infarction, HF = heart failure, HR = hazard ratio, CI = confidence interval, cs-cTnI = troponin I measured by contemporary-sensitivity assay, hs-cTnI = troponin I measured by high-sensitivity assay, ss-cTnI = troponin I measured by super-sensitivity assay, ns = not significant

**Table S3: C-Statistics and Integrated Discrimination Improvement for Baseline Troponin Assessed by Contemporary-Sensitivity, High-Sensitivity and Super-Sensitivity Assays for Various Endpoints after Adjustment for the Framingham Risk Score**

| **Event** | **Troponin** | **C old** | **C new** | **p value** | **IDI** | **p value** |
| --- | --- | --- | --- | --- | --- | --- |
| **MACE** | cs-cTnI | 0.8287 | 0.8292 | ns | 0.0004 | ns |
|  | hs-cTnI | 0.8287 | 0.8290 | ns | 0.0012 | ns |
|  | ss-cTnI | 0.8287 | 0.8302 | ns | 0.0032 | 0.0072 |
| **CVD** | cs-cTnI | 0.8419 | 0.8427 | 0.0229 | 0.0008 | ns |
|  | hs-cTnI | 0.8419 | 0.8418 | ns | 0.0003 | ns |
|  | ss-cTnI | 0.8419 | 0.8426 | ns | 0.0019 | ns |
| **MI** | cs-cTnI | 0.8464 | 0.8487 | 0.0303 | 0.0013 | ns |
|  | hs-cTnI | 0.8464 | 0.8460 | ns | 0.0010 | ns |
|  | ss-cTnI | 0.8464 | 0.8489 | ns | 0.0077 | 0.0013 |
| **HF** | cs-cTnI | 0.8217 | 0.8223 | ns | 0.0002 | ns |
|  | hs-cTnI | 0.8217 | 0.8247 | 0.0216 | 0.0027 | 0.0137 |
|  | ss-cTnI | 0.8217 | 0.8266 | 0.0061 | 0.0072 | 0.0007 |
| **Stroke** | cs-cTnI | 0.8260 | 0.8260 | ns | -0.0001 | ns |
|  | hs-cTnI | 0.8260 | 0.8261 | ns | -0.0001 | ns |
|  | ss-cTnI | 0.8260 | 0.8270 | ns | -0.0002 | ns |
| **Death** | cs-cTnI | 0.8174 | 0.8186 | 0.0008 | 0.0003 | ns |
|  | hs-cTnI | 0.8174 | 0.8173 | ns | -0.0002 | ns |
|  | ss-cTnI | 0.8174 | 0.8176 | ns | 0.0002 | ns |

MACE = major adverse cardiac events, CVD = cardiovascular disease, MI = myocardial infarction, HF = heart failure, cs-cTnI = troponin I measured by contemporary-sensitivity assay, hs-cTnI = troponin I measured by high-sensitivity assay, ss-cTnI = troponin I measured by super-sensitivity assay, IDI = integrated discrimination improvement, ns = not significant
